# Sprains, Strains and Growing Pains: Managing Cognitive Bias to Facilitate Timely Diagnosis in Pediatric Sports Medicine

**DOI:** 10.3390/children12060784

**Published:** 2025-06-16

**Authors:** Parker Scott, Leslie Sim, David Soma, Bo E. Madsen, Bjorg Thorsteinsdottir

**Affiliations:** 1 Department of Biology, St. Olaf College, Northfield, MN 55057, USA; scott27@stolaf.edu; 2 Department of Psychiatry and Psychology, Mayo Clinic, Rochester, MN 55905, USA; sim.leslie@mayo.edu; 3 Department of Pediatric and Adolescent Medicine, Mayo Clinic, Rochester, MN 55905, USA; soma.david@mayo.edu; 4 Department of Orthopedic Surgery, Mayo Clinic, Rochester, MN 55905, USA; 5 Department of Emergency Medicine, Mayo Clinic, Rochester, MN 55905, USA; madsen.bo@mayo.edu; 6 Department of Community Internal Medicine, Mayo Clinic, Rochester, MN 55905, USA

**Keywords:** diagnoses and examination, cognitive bias, medical decision making, return-to-sport, adolescents, sports medicine

## Abstract

Background: Diagnostic delay and error represent pervasive problems in healthcare with grave implications for treatment and prognosis. Though characteristic of human cognition, cognitive biases commonly contribute to delays in the physician decision-making process, particularly in atypical or complex presentations in youth. Methods: We present a case series of three adolescent athletes with varied clinical presentations whose diagnostic conceptualization and treatment were delayed in part due to cognitive biases with consequences for overall health and development, as well as return to sport. Results: The first case depicts how an atypical presentation of celiac disease was attributed to growing pains, illustrating the contribution of anchoring bias and confirmation bias in medical decision making. The second case represents the misattribution of chronic exertional compartment syndrome pain to growing pains and post-exercise soreness, highlighting the influence of ascertainment bias on the initial misdiagnosis. The third case describes how a vertebral mass was misdiagnosed as a left shoulder strain from weightlifting, depicting the contribution of anchoring bias and ascertainment bias in medical decision making. Conclusions: Early recognition of cognitive biases, including confirmation bias, anchoring bias, and ascertainment bias, is crucial for improving medical decision making, particularly in cases of rare or atypical presentations, reducing unnecessary diagnostic delays, and setting more realistic patient expectations. Through discussion of these cases, we highlight concrete steps to manage bias to facilitate timely diagnosis within the primary care and sports medicine setting.

## 1. Introduction

Diagnostic error and delay are a pervasive challenge in the field of medicine [1]. In adolescent athletes, diagnostic error and subsequent delay in diagnosis may have profound consequences for their overall health and development, as well as athletic aspirations. Yet complex presentations in this age group can pose a unique challenge to detection. Depending on age and pubertal development, youth may present with injuries commonly seen in both skeletally immature young children and more skeletally mature adults. For example, a 13-year-old athlete could present with either a fracture (more common in preadolescent youth) or a sprain of the ankle ligaments (more common in post-pubertal adolescents).

In addition, adolescents may also complain of vague symptoms and pain that can be dismissed as normal developmental changes or experience and describe pain differently [2]. For example, two athletes can experience an ACL tear differently, where one continues to play, and the other athlete comes to the emergency department in excruciating pain. Moreover, common patient characteristics and symptom presentations among athletes may obscure more rare and atypical conditions. Unfortunately, when clinicians overlook underlying conditions, it can perpetuate delays in care and return to sports activities with potential enduring implications for adolescents’ social and emotional development.

Despite advances in diagnostic technology and medical knowledge, diagnosis is often delayed by limited time to evaluate complex problems and overlapping disease presentations, among other variables inherent to the healthcare encounter and presenting problem [3]. Lesser known but potentially modifiable factors that contribute to delayed diagnosis are cognitive biases among clinicians. As with all humans, clinicians are naturally predisposed to forming a premature or undue impression that is not factually based [4]. Confirmation bias, anchoring bias, and ascertainment bias represent common types of biases that can influence clinical decision making. These types of thinking errors are common and can seriously affect clinical decisions in fast-moving sports settings, as represented in the cases below. These three cases illustrate how these mental shortcuts can cause real-world issues and what we can learn from those situations.

## 2. Case Series

### 2.1. Case 1

“John” is an athletic and physically active 10-year-old boy who faced a significant setback when he developed debilitating mid-thoracic back pain without a clear precipitant. Prior to the onset of his pain, sports such as soccer, handball, and skateboarding were central to his daily life and social interactions. These activities not only provided physical exercise but also fostered relationships with his friends and teammates. The onset of this debilitating pain forced him to withdraw from activities, severely impacting his social life. Unable to participate in sports, he became increasingly sedentary and isolated, a situation he found frustrating and demoralizing.

The road to diagnosis was lengthy and challenging, as his symptoms, especially severe mid-thoracic back pain, suggested various conditions. During initial appointments in primary care, his pain was attributed to growing pains that would resolve without intervention. With persistence and worsening of his symptoms, his physician obtained spinal X-rays that did not show any diagnostic abnormalities, and he was referred to physical therapy without improvement in his symptoms. With persistence of his symptoms, and his mother’s perseverance, he was referred to several different specialists over the coming years, including rheumatology, and had more detailed imaging studies, including MRI of the thoracic spine, again without any identified diagnostic abnormality. The family also sought chiropractic treatment without relief of his symptoms. In the absence of identifiable pathology, psychiatric testing was also conducted, ruling out mental health concerns such as anxiety and depression as causative. After close to four years without a diagnosis, during an admission for severe abdominal pain, extensive blood work revealed a severe vitamin D deficiency. Subsequent serology and genetic testing revealed celiac disease, a condition associated with a wide range of nearly 300 symptoms, which presented itself atypically in his case. Indeed, upon further review of his history, he had suffered stomach aches and gastrointestinal problems including intermittent diarrhea since early childhood. After being diagnosed, he began a gluten-free diet and is now thriving socially, emotionally, and physically without debilitating back pain and other symptoms he once experienced. John’s case highlights how celiac disease can mimic musculoskeletal conditions and the need for physicians to broaden their evaluation in the face of ongoing diagnostic uncertainty [5,6]

### 2.2. Case 2

“Lewis” is a 13-year-old boy who was an accomplished soccer player, selected at an early age to join a prestigious soccer academy in Europe with goals of someday playing professionally. His journey, however, was disrupted by debilitating bilateral exertional calf pain that developed following a mild tear of his Achilles tendon, resulting in a two-month break from both soccer and workouts. Upon returning to training, he developed pain in both calves, initially dismissed as typical post-rest soreness. As the discomfort persisted, it was attributed to growing pains with shortened muscles and he was referred to physical therapy. Despite diligent adherence to physical therapy and recommended stretches at the academy, his symptoms worsened. Running and jumping caused intense bilateral calf pain, escalating during vigorous activities, forcing him to cease at 20–30 min with a prolonged recovery period. Eventually, even moderate exertion triggered discomfort, affecting his ability to walk long distances or engage in uphill biking.

After failing training modifications, he sought help from an osteopath who thought slight problems with balance due to flat feet and eyesight might be to blame. Trials of shoe inserts and glasses were tried, as well as more physical therapy. Subsequently, a sports medicine specialist ordered a detailed movement analysis indicating shortened muscles. However, despite diligent adherence to the exercises over a 6-week period, he had no improvement. Lewis was then referred to neurology and embarked on an extensive nine-month diagnostic investigation. This included MRI of both calves, normal blood counts and a comprehensive metabolic panel except for mild elevation of CK, normal muscle biopsies, genetic tests ruling out myopathies, and unremarkable nerve conduction studies except for minor delays in one peroneal nerve. Yet his condition remained undiagnosed and untreated. A Doppler study to examine popliteal entrapment, while inconclusive, led to recommendations for further dynamic Doppler assessments after activity, MRI scans of both hips, and a rheumatological evaluation.

Throughout this prolonged medical journey spanning nearly three years and prolonged by the COVID-19 pandemic, he received neither a definitive diagnosis nor an improvement in symptoms. Medical professionals speculated that he might outgrow his condition, as he had approximately 5–10 cm to reach his adult height. Meanwhile, the lengthy ordeal resulted in losing his place in the academy and ceasing soccer altogether, adversely impacting his physical and emotional health.

Despite these challenges, he remained socially integrated and maintained connections with friends from soccer and other aspects of his life. However, the emotional toll was considerable, leading to a referral to a psychiatrist who concluded that his symptoms were not attributable to any form of performance anxiety or depression as some physicians had started to speculate. A chance visit with a family friend with a medical background eventually led to a second opinion at a tertiary referral center, where the diagnosis of exertional compartment syndrome was suggested and later confirmed. After a successful operation, he was able to return to playing soccer, albeit not at his previous level because of lost training during a crucial time in his physical development. Lewis’s case stresses the need to consider compartment syndrome earlier in the course of diagnostic evaluation in teens who are competitive athletes.

### 2.3. Case 3

“Michael” is a 16-year-old boy who ran track and field and played basketball while in high school. When he was 15, he began experiencing left shoulder pain. This pain was originally attributed to his participation in weightlifting. However, he shortly thereafter noticed some pins-and-needles pain along the posterior lateral aspect of his left arm. He reached out to his local pediatrician, and due to the presence of pain and symptoms in the left chest out to the arm, he was referred to cardiology where he underwent an evaluation that was unremarkable, including an EKG and echocardiogram. He was also evaluated by pediatric gastroenterology without any notable findings.

While he continued participating in basketball, he struggled to do so related to ongoing shoulder and arm pain. Consequently, he was evaluated in sports medicine and was prescribed physical therapy, a treatment course that resulted in limited improvement. He later underwent an MRI of the left shoulder, which was unremarkable, but pain continued to be attributed to shoulder and pectoralis muscle injury. In spite of the pain, the patient tried to make it through the basketball season, which was cut short due to the COVID-19 pandemic. While he attempted some of his own exercise and home therapy, his symptoms worsened. Later, his symptoms evolved to include increased thoracic back pain, more notably on the left side. His pain worsened when he ran using arm motions, sneezed, or performed certain motions of the shoulder and neck. His clinician referred him to a pain specialist.

Instead, a year after his original symptoms started, he sought a second opinion for a multidisciplinary evaluation within a pediatrics department at a large medical center. In the triage process, he was rejected for this evaluation due to his prior diagnosis of chronic pain. In response, the family requested that a pediatric sports medicine physician take a second look at the records, particularly as the symptoms started in response to an injury. The pediatric sports medicine physician felt that a second opinion was warranted and recommended an additional MRI to include the chest/thoracic spine, which discovered a lobulated enhancing mass centered around the T2 vertebral body with extension into the adjacent tissues. The biopsy was consistent with osteoblastoma. Within a month of his second opinion, he proceeded with a planned two-stage tumor resection from his thoracic spine. He first underwent an embolization procedure as an outpatient, followed by anterior surgery to resect his T2 mass, as well as posterior decompression, tumor resection, and instrumented fusion.

The patient recovered successfully from this extensive operation. However, the emotional impact of the year-long journey was significant. Following surgery, he experienced severe hand pain that was unremitting with oxycodone and lidocaine patches. In response, he showed poor pain coping including perseverating on the pain at the exclusion of other activities in his life and marked behavioral outbursts. He also showed a functional gait and guarding in his left arm. Though he had been cleared to return to school, Michael protested returning and sought additional prescriptions for narcotic medication.

Upon evaluation, his surgeon felt there was a strong emotional overlay that was contributing to his ongoing pain and poor recovery. He suspected the medication seeking, functional limitations, and symptoms, as well as pain behaviors, were in response to the significant emotional impact of the year-long journey of unexplained symptoms. To allay his fears and support his recovery, his surgeon had reassured Michael that his recovery was progressing well. He discouraged narcotic medications and prescribed gabapentin for post-surgical neuritis and strongly encouraged him to return to normal developmental activities, including school and physical activities. With parental support for functioning, 6 months later, Michael took a position as a camp counselor and 2 years later, he was attending college and back to running and weightlifting.

Michael’s case highlights how a label of chronic pain can mask more rare conditions, as well as the need to rule out other conditions prior to making this diagnosis. It also demonstrates that once the diagnosis of chronic pain is made, it can preclude future investigations, as was seen in the triage process which initially rejected the opportunity for a second opinion. It also highlights the importance of ensuring new information is analyzed to see if it correlates with prior diagnoses. Similar to case 2, Michael’s case also highlights the impact of the COVID-19 pandemic on reducing access to care and exacerbating delays in diagnosis and treatment.

## 3. Discussion

All of these cases illustrate diagnostic misdirection in the face of underlying stealth processes, circumstances in which the underlying pathologies differed from classic presentations, contributing to delayed diagnosis. These misdiagnoses underscore the need for pediatricians and sports medicine clinicians to maintain the possibility of rare or atypical pathologies in the differential diagnosis.

The first case, for example, illustrates how vague symptoms of a nutritional deficiency related to an autoimmune disorder can pose unique obstacles to timely diagnosis. Celiac disease remains a challenging condition to identify, with most patients diagnosed in adulthood [7]. Musculoskeletal symptoms, including low bone mineral density, are common among patients with celiac disorder, but may not be commonly thought of in this context [5]. One study found a higher prevalence of celiac disorder in young adults presenting to a sports medicine clinic with stress fractures, suggesting that a young person presenting with a stress fracture should be tested for celiac disease [5,8]. Though thoracic back pain is not a common site of growing pains, his pain was originally attributed to this condition, partly because he was a very tall boy going through his adolescent growth spurt during this time. This attribution is a prime example of anchoring bias in medical decision making. This cognitive bias arises from tendencies to overly rely on initial impressions in the diagnostic process and not adjusting from this conceptualization in the face of newly available information [9]. The term “growing pains” is a diagnosis of exclusion and, unfortunately, prevented a search for new or relevant information in spite of the persistence of the pain without clarity. Meanwhile, John underwent several imaging studies and referrals, accumulating negative results that led to consistent dismissal and minimization of his symptoms until his eventual diagnosis. None of the clinicians made the connection between the musculoskeletal symptoms and his long-standing gastrointestinal problems.

The second case depicts how ascertainment and confirmation bias can result in diagnostic delay. Ascertainment bias happens when the clinician’s thinking is pre-shaped by expectations, leading to underassessment or overassessment of the patient’s conditions [9]. This cognitive bias tends to occur when associating specific diagnoses with stereotypical patient characteristics. As a young, elite athlete, it would be common to assume that the patient’s lower-extremity pain has stemmed from post-exercise soreness, a frequently occurring condition in athletes engaging in high-intensity training. It would be further understandable that a treatment plan would be based on experience with patients with similar characteristics. Though his pain worsened, it is likely that confirmation bias functioned to maintain the erroneous diagnostic conceptualization and treatment plan. Confirmation bias occurs when there is a search for evidence that supports the clinician’s diagnostic conceptualization while ignoring symptoms or signs that do not support this hypothesis [9], for example, noticing a sign that supports a specific diagnosis on imaging, but not examining confounding features that might assist with creating a broader differential diagnosis. In chronic exertional compartment syndrome (CECS), there are often no neurovascular deficits or permanent tissue damage occurring in the compartment. In addition, symptoms are transient and physical examination findings during non-exertion periods are frequently inconclusive. As such, diagnosing CECS can be unusually challenging. As such, on average, there is a 22-month delay in the diagnosis of CECS [10]. The nearly 2-year delay has significant implications not only for missed athletic training and development but also for a young athlete’s quality of life. The delay in diagnosis, coupled with its relatively common occurrence, highlights the importance for clinicians to more readily consider CECS as a possible diagnosis that could have a negative effect on an individual’s participation in sports [11]. This case highlights the need to revise the diagnostic conceptualization when treatments for suspected conditions do not alleviate the problem.

The third case represents how ascertainment bias coupled with anchoring bias can foster diagnostic delay with severe consequences for physical development and function. Because of the rarity and nonspecific symptoms of muscular pathology which characterize this condition in pediatric patients, it is common to apply adult concepts to children, a possible contribution to missed diagnoses in Michael’s case, a prime example of ascertainment bias. With regard to anchoring bias, the “anchor” in this case might have been weightlifting, to which the original symptoms were attributed. Because the initial pain was in the shoulder, it was logical for the clinician to consider that was the source of the pain. Yet when the youth did not respond to treatment and the symptoms changed, the diagnostic conceptualization was not modified. A second opinion was extremely beneficial in his case as it provided a fresh perspective to identify new clues to his presentation.

Though these cases highlight delayed diagnoses and potential biases, it is important to note that they are limited by retrospective chart review methods, which are subject to missing information. In addition, the biases described in the report were inferred and not identified by empirical methodology. Future research is necessary on interventions to reduce the impact of cognitive bias on medical decision making in pediatrics and pediatric sports medicine settings.

### Reducing Diagnostic Error Through Management of Cognitive Biases

While diagnostic error and delay represent a pervasive problem across the field of medicine, there are ways to manage and prevent them [1]. Debiasing strategies have been explored, such as slowing down and being more deliberate in one’s reasoning, collecting evidence to support a diagnosis that is opposite to one’s initial impressions, and using checklists and decision support tools integrated into the medical record [12]. As empirical support for these strategies in reducing cognitive biases is mixed, cognitive biases can also be minimized through clinical strategies such as performing a comprehensive assessment of the patient, considering factors such as activity levels, social dynamics, familial history, and relevant genetic information and being mindful of the potential for stereotyping based on patient characteristics [13].

Comprehensive assessment may also include maintaining open communication with the patient and caregiver. It is essential to maintain an integrated and ongoing conversation as it may uncover subtle abnormalities that may not be evident in standard laboratory tests but are indicative of underlying health issues. Moreover, sports medicine clinicians can take a patient-centered perspective, which involves being patient and curious, as well as being careful to not hastily attribute physical symptoms to psychiatric disorders when diagnosing complex cases [14]. Though it is common for psychiatric symptoms to accompany these cases, it is important to recognize that they are frequently secondary to the physical symptoms and impairments in function. In addition, healthcare clinicians should collaborate with specialists to integrate diverse findings for accurate diagnosis and continuity of care [15]. Finally, clinicians must beware of carrying forward prior diagnoses without reviewing the findings.

Several additional key elements have potential to minimize bias and increase diagnostic accuracy. First, when applying a treatment for a suspected injury or condition, clinicians should outline an expected course with a follow-up plan in some capacity. If the expected course is different or on follow-up the treatment is not effective, then a reassessment of diagnosis and broadening of differential diagnosis should be considered. Second, every medical condition has a typical narrative. When gathering key elements of history, it is critical to analyze those elements that do not fit the narrative. Those key elements should prompt consideration for additional testing or evaluation. Third, most competitive athletes have a strong desire for participation in the sports/activities they enjoy. Individuals who continue to try and participate but fail despite following the recommended treatment indicate a need for reassessment. Though rare conditions are commonly not diagnosed at the time of the first medical evaluation, through a careful and intentional approach, such diagnoses can still be made.

In addition, a cornerstone to mitigating cognitive bias in medicine is whole-person care (WPC), a patient-centered approach that considers the intersecting biological, psychological, social, and contextual domains of a person’s life. WPC contrasts from the reductionistic focus on organ systems and views health and disease within a system of interconnected biological, behavioral, social, and environmental factors. In all of these cases, the undiagnosed medical condition had an impact on the patients’ behavior, functioning, and social life. Key components of WPC include synthesizing diverse information, incorporating multiple follow-up consultations over time, and utilizing diverse treatment modalities within a collaborative team-based approach [14]. As the incidence of multimorbidity and complex diagnoses rises, this approach becomes essential [16]. In each of these cases, underlying problems were revealed after second opinions from novel specialties that included regular follow-up, after establishing a trusting doctor–patient relationship that incorporated the patients’ and families’ concerns and perspectives.

Complex cases often spur clinicians to inadvertently narrow their focus on the perceived problem (e.g., chronic pain) or explanation (e.g., physical injury), neglecting the broader impacts of life experiences, relationships, or context [17]. Tunnel vision in these presentations can result in care becoming ‘stuck’, requiring a more comprehensive perspective to effectively address it. In the management of complex cases, clinicians will need to collaborate effectively and utilize the expertise from other specialized healthcare professionals. See Table 1 for more information on strategies to prevent cognitive bias.

Healthcare clinicians are human and therefore subject to the all-too-common influence of cognitive bias in medical decision making [18]. Early recognition of biases, including confirmation bias, anchoring bias, and ascertainment bias, among clinicians is crucial for improving medical decision making, reducing unnecessary diagnostic delays, and setting more realistic patient expectations. Additionally, education on bias and ways to minimize its harms should be part of health system-wide efforts to standardize knowledge in this area and to mitigate the influence of cognitive biases on medical decisions [19]. Clinicians are encouraged to slow down, reflect on their decision making, try to disconfirm their original hypotheses, and use diagnostic checklists and tools to support decision making. They can also integrate a comprehensive assessment that emphasizes the clinician–patient relationship and integrates a longitudinal assessment through follow-up visits. When in doubt, clinicians can also consult other specialists.

## 4. Conclusions

These cases of adolescent athletes highlight diagnostic delays that contributed to worsening symptoms, emotional stress for the patient, missed treatment windows, delayed return to sport, and attenuated athletic development. However, they also show how keeping an open mind and checking our biases can make a real difference in diagnosing young athletes, helping them return to sport and life. While the diagnostic delays highlighted in the cases are a common occurrence in medicine, they can be minimized through awareness of cognitive bias during the diagnostic decision-making process, open communication with caregivers and the patient, and effective collaboration with specialists.

## Figures and Tables

**Table 1:** Strategies to mitigate diagnostic error and delay.

Initial presentation—Approach the problem with curiosity and an open mind.
Listen carefully to the patient’s description of their symptoms, how they started and how they have evolved. Avoid the temptation to ask leading or clarifying questions about other symptoms to confirm your initial hunch for a diagnosis. Beware that sometimes patients may wrongfully attribute symptoms to a minor trauma. Ask yourself does the mechanism of injury fit with the presenting symptoms. Recognize that the site of the pain is not always the source of the pain. Perform a careful and thorough physical examination. Is there a need for imaging studies or does the physical examination suffice? Review other systems to look for clues to other potential underlying disease processes.
Follow up—Partner with your patient and their parents.
Share your top differential diagnoses with the patient and parents and explain to them the expected symptom evolution and recovery time. Recommend a return visit if the symptoms worsen or do not follow the expected time frame. When symptoms persist without clarity, additional testing is indicated. When the symptoms do not respond to initial treatment, revise diagnosis and treatment plan. Outline red flags that require a more urgent reassessment or imaging. Keep an open line of communication.
Referral and reassessment—Is the patient on the right track?
Is the adolescent motivated to return to their sport/normal activities and trying hard? ◦ If yes, troubleshoot why they might be failing—what are you missing? ◦ If no, ask why might that be? Activate the multidisciplinary team (i.e., PT, nursing, nutrition). If necessary, support the need for a second opinion.
Other considerations
Is there concern for overtraining and stress injury? Is there concern for nutritional deficiency and/or eating disorder? Has there been a dramatic change in the adolescent’s mood? Could there be psychosocial factors other than the injury underlying the presentation (e.g., bullying, substance use)?

## Data Availability

The datasets presented in this article are not readily available because they represent protected health information. Requests to access the datasets should be directed to the corresponding author who can provide de-identified data.
